# 4-(4-Chloro­phen­yl)-1-(2-hydr­oxy-2,2-di­phenyl­acet­yl)thio­semicarbazide

**DOI:** 10.1107/S1600536808035964

**Published:** 2008-11-13

**Authors:** Kayed A. Abu-Safieh, Monther A. Khanfar, Klaus Eichele, Basem Fares Ali

**Affiliations:** a Department of Chemistry, The Hashemite University, Zarqa, Jordan; b Department of Chemistry, The University of Jordan, Amman 11942, Jordan; c Institut für Anorganiche Chemie, Universität Tübingen, Auf der Morgenstelle 18, 72076 Tübingen, Germany; d Department of Chemistry, Al al-Bayt University, Mafraq 25113, Jordan

## Abstract

The asymmetric unit of the title compound, C_21_H_18_ClN_3_O_2_S, contains two mol­ecules in which the bond lengths and angles are almost identical. Intra­molecular N—H⋯S hydrogen bonds result in the formation of two five-membered rings. In the crystal structure, inter­molecular N—H⋯O hydrogen bonds link the mol­ecules into centrosymmetric dimers; these dimers are linked *via* inter­molecular O—H⋯S hydrogen bonds, leading to infinite corrugated layers parallel to the *bc* plane through *R*_2_^2^(16) ring motifs.

## Related literature

For a related structure, see: Ergenç *et al.* (1992 ▶). For general background, see: Jalilian *et al.* (2000 ▶); John (1998 ▶); Kucukguzel *et al.* (2006 ▶); Shen *et al.* (1998 ▶); Singh *et al.* (2005 ▶). For ring motifs, see: Bernstein *et al.* (1995 ▶).
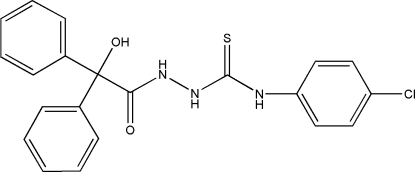


## Experimental

### 

#### Crystal data

C_21_H_18_ClN_3_O_2_S
*M* *_r_* = 411.89
Monoclinic, 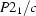

*a* = 14.1039 (19) Å
*b* = 18.1566 (19) Å
*c* = 16.9108 (19) Å
β = 114.509 (10)°
*V* = 3940.3 (9) Å^3^
*Z* = 8
Mo *K*α radiation
μ = 0.32 mm^−1^
*T* = 173 (2) K
0.9 × 0.4 × 0.4 mm

#### Data collection

Bruker P4 diffractometer
Absorption correction: multi-scan (*SADABS*; Bruker, 2005 ▶) *T*_min_ = 0.837, *T*_max_ = 0.879
20863 measured reflections
9027 independent reflections
6867 reflections with *I* > 2σ(*I*)
*R*_int_ = 0.029
2 standard reflections every 98 reflections intensity decay: none

#### Refinement

*R*[*F* ^2^ > 2σ(*F* ^2^)] = 0.041
*wR*(*F* ^2^) = 0.102
*S* = 1.02
9027 reflections
532 parameters
H atoms treated by a mixture of independent and constrained refinement
Δρ_max_ = 0.91 e Å^−3^
Δρ_min_ = −0.82 e Å^−3^

### 

Data collection: *XSCANS* (Bruker, 1996 ▶); cell refinement: *XSCANS*; data reduction: *SHELXTL* (Sheldrick, 2008 ▶); program(s) used to solve structure: *SHELXS97* (Sheldrick, 2008 ▶); program(s) used to refine structure: *SHELXL97* (Sheldrick, 2008 ▶); molecular graphics: *SHELXTL*; software used to prepare material for publication: *SHELXTL*.

## Supplementary Material

Crystal structure: contains datablocks I, New_Global_Publ_Block. DOI: 10.1107/S1600536808035964/hk2559sup1.cif

Structure factors: contains datablocks I. DOI: 10.1107/S1600536808035964/hk2559Isup2.hkl

Additional supplementary materials:  crystallographic information; 3D view; checkCIF report

## Figures and Tables

**Table 1:** Hydrogen-bond geometry (Å, °)

*D*—H⋯*A*	*D*—H	H⋯*A*	*D*⋯*A*	*D*—H⋯*A*
O1—H1*O*⋯S2^i^	0.84	2.44	3.2242 (13)	156
N1—H1*N*⋯S1	0.89 (2)	2.44 (2)	2.9075 (16)	113.5 (17)
N2—H2*N*⋯O5^ii^	0.81 (2)	2.12 (2)	2.870 (2)	155 (2)
N3—H3*N*⋯O5^ii^	0.88 (2)	2.17 (2)	3.003 (2)	156.1 (18)
O4—H4*O*⋯S1^iii^	0.84	2.51	3.2707 (13)	151
N4—H4*N*⋯S2	0.88 (2)	2.50 (2)	2.9569 (16)	113.5 (17)
N5—H5*N*⋯O2^iv^	0.90 (2)	1.96 (2)	2.776 (2)	149 (2)
N6—H6*N*⋯O2^iv^	0.89 (2)	2.02 (2)	2.842 (2)	154 (2)

Symmetry codes: (i) 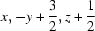; (ii) 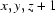; (iii) 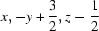; (iv) 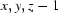.

**Fig. 1.**
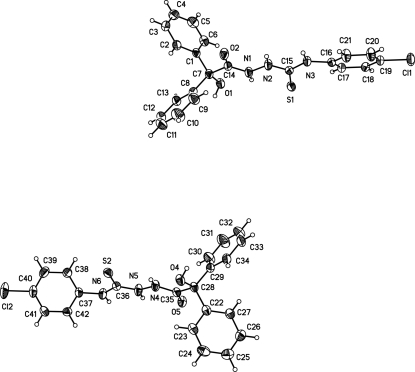
The molecular structure of the title molecule, with the atom-numbering scheme. Displacement ellipsoids are drawn at the 50% probability level.

**Fig. 2.**
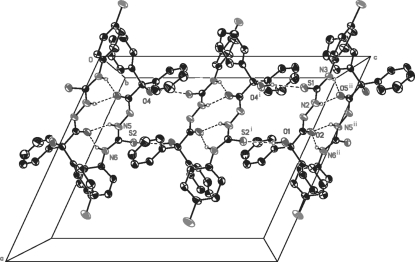
A partial packing diagram. Hydrogen bonds are shown as dashed lines [symmetry codes: (i) x, 3/2 - y, z + 1/2; (ii) x, y, z + 1]. H atoms not involved in hydrogen bonding are omitted for clarity.

**Fig. 3.**
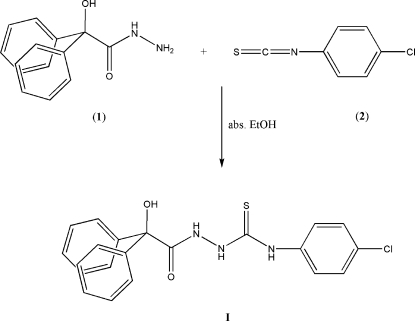
A schematic representation of the reaction that afforded the title compound.

## supplementary crystallographic information

### Comment

Thiosemicarbazides have received special interest for their potential biological activities (Kucukguzel *et al.*,  2006; Singh *et al.*,  2005). They have also received considerable attention because of the possibility of their use as intermediates in the synthesis of many biologically active heterocyclic compounds such as 1,2,4-triazole derivatives (Ergenç *et al.*, 1992), 1,3,4-thiadiazoles (Jalilian *et al.*,  2000) and many others. As ligands, thiosemicarbazides are useful bidentate ligands (*S*- and *N*- donors) for transition metal ions and their complexes possess many biological activities (Shen *et al.*,  1998). The title compound was synthesized as an intermediate for biologically active 1,2,4-triazole derivative (Ergenç *et al.*,  1992). We report herein its crystal structure.

The asymmetric unit of the title compound contains two independent thiosemicarbazide molecules (Fig 1), where the bond lengths and angles are almost identical (Table 1). In both molecules, the linking C-N-N-C-N units are delocalized and flattened. The C-S and C-O bonds both show the double bond character, while the C-N and N-N bonds in the linking units imply significant electron delocalization. As a result of conjugation, O2-C14 [1.241 (2) Å] and O5-C35 [1.242 (2) Å] bonds are longer than the normal value of 1.20 Å (John, 1998), while N1-C14 [1.323 (2) Å] and N4-C35 [1.327 (2) Å] bonds are in accordance with the C-N double bond length (1.32 A°; John, 1998) and shorter than the C-N single bond length (1.475 A°; John, 1998). The sum of the bond angles around N1, N2, N3, C14, C15 and N4, N5, N6, C35, C36 atoms are about 360°, which implies *sp^2^* hybridization for these atoms. The thiourea group is approximately planar. The intramolecular N-H···S hydrogen bonds (Table 2) result in the formation of two five-membered rings (S1/N1/N2/C15/H1N) and (S2/N4/N5/C36/H4N).

In the crystal structure, intermolecular N-H···O hydrogen bonds (Table 2) link the molecules into centrosymmetric dimers (Fig. 2), in which they are also linked to the other dimers via intermolecular O-H···S hydrogen bonds (Table 2) leading to infinite corrugated layers parallel to the bc plane through R_2_^2^(16) ring motifs (Bernstein *et al.*,  1995).

### Experimental

The title compound was synthesized according to the literature method (Ergenç *et al.*,  1992) by the reaction of equimolar amounts of 2-hydroxy-2,2-diphenyl- acetohydrazide, (1), and 1-chloro-4-isothiocyanatobenzene, (2), (Fig. 3). Crystals suitable for X-ray analysis were obtained by recrystallization from a methanol solution at room temperature.

### Refinement

H1N, H2N, H3N, H4N, H5N and H6N atoms (for NH) were located in difference syntheses and refined isotropically [N-H = 0.81 (2)-0.90 (2) Å and U_iso_(H) = 0.032 (5)-0.046 (7) Å^2^]. The remaining H atoms were positioned geometrically, with O-H = 0.84 Å (for OH) and C-H = 0.95 Å for aromatic H, respectively, and constrained to ride on their parent atoms with U_iso_(H) = 1.2U_eq_(C,O).

### Crystal data

**Table:**

C_21_H_18_ClN_3_O_2_S	*F*_000_ = 1712
*M**_r_* = 411.89	*D*_x_ = 1.389 Mg m^−^^3^
Monoclinic, *P*2_1_/*c*	Mo *K*α radiation λ = 0.71073 Å
Hall symbol: -P 2ybc	Cell parameters from 51 reflections
*a* = 14.1039 (19) Å	θ = 4.9–12.6º
*b* = 18.1566 (19) Å	µ = 0.32 mm^−^^1^
*c* = 16.9108 (19) Å	*T* = 173 (2) K
β = 114.509 (10)º	Prism, colorless
*V* = 3940.3 (9) Å^3^	0.9 × 0.4 × 0.4 mm
*Z* = 8	

### Data collection

**Table:**

Bruker P4 diffractometer	*R*_int_ = 0.029
Radiation source: fine-focus sealed tube	θ_max_ = 27.5º
Monochromator: graphite	θ_min_ = 2.2º
*T* = 173(2) K	*h* = −18→1
ω scans	*k* = −23→23
Absorption correction: multi-scan(SADABS; Bruker, 2005)	*l* = −20→21
*T*_min_ = 0.837, *T*_max_ = 0.879	2 standard reflections
20863 measured reflections	every 98 reflections
9027 independent reflections	intensity decay: none
6867 reflections with *I* > 2σ(*I*)	

### Refinement

**Table:**

Refinement on *F*^2^	Hydrogen site location: inferred from neighbouring sites
Least-squares matrix: full	H atoms treated by a mixture of independent and constrained refinement
*R*[*F*^2^ > 2σ(*F*^2^)] = 0.041	*w* = 1/[σ^2^(*F*_o_^2^) + (0.0394*P*)^2^ + 1.5051*P*] where *P* = (*F*_o_^2^ + 2*F*_c_^2^)/3
*wR*(*F*^2^) = 0.102	(Δ/σ)_max_ = 0.001
*S* = 1.02	Δρ_max_ = 0.91 e Å^−^^3^
9027 reflections	Δρ_min_ = −0.82 e Å^−^^3^
532 parameters	Extinction correction: SHELXL, Fc^*^=kFc[1+0.001xFc^2^λ^3^/sin(2θ)]^-1/4^
Primary atom site location: structure-invariant direct methods	Extinction coefficient: 0.0023 (2)
Secondary atom site location: difference Fourier map	

### Special details

**Table:**

Geometry. All e.s.d.'s (except the e.s.d. in the dihedral angle between two l.s. planes) are estimated using the full covariance matrix. The cell e.s.d.'s are taken into account individually in the estimation of e.s.d.'s in distances, angles and torsion angles; correlations between e.s.d.'s in cell parameters are only used when they are defined by crystal symmetry. An approximate (isotropic) treatment of cell e.s.d.'s is used for estimating e.s.d.'s involving l.s. planes.
Refinement. Refinement of *F*^2^ against ALL reflections. The weighted *R*-factor *wR* and goodness of fit *S* are based on *F*^2^, conventional *R*-factors *R* are based on *F*, with *F* set to zero for negative *F*^2^. The threshold expression of *F*^2^ > σ(*F*^2^) is used only for calculating *R*-factors(gt) *etc*. and is not relevant to the choice of reflections for refinement. *R*-factors based on *F*^2^ are statistically about twice as large as those based on *F*, and *R*- factors based on ALL data will be even larger.

### Fractional atomic coordinates and isotropic or equivalent isotropic displacement parameters (Å^2^)

**Table:**

	*x*	*y*	*z*	*U*_iso_*/*U*_eq_	
Cl1	−0.35372 (4)	0.47876 (3)	0.91532 (4)	0.04719 (14)	
S1	0.10518 (4)	0.53220 (3)	0.84004 (3)	0.03220 (11)	
O1	0.40810 (11)	0.59866 (7)	0.86727 (8)	0.0351 (3)	
H1O	0.4136	0.5983	0.8197	0.053*	
O2	0.34635 (10)	0.74781 (7)	0.97416 (9)	0.0372 (3)	
N1	0.26430 (12)	0.64596 (9)	0.90301 (10)	0.0316 (3)	
H1N	0.2627 (16)	0.6047 (12)	0.8741 (14)	0.041 (6)*	
N2	0.18196 (12)	0.65529 (9)	0.92635 (11)	0.0327 (4)	
H2N	0.1839 (18)	0.6904 (13)	0.9562 (15)	0.046 (7)*	
N3	0.03119 (11)	0.62184 (8)	0.93040 (10)	0.0280 (3)	
H3N	0.0423 (15)	0.6629 (11)	0.9608 (13)	0.032 (5)*	
C1	0.53250 (13)	0.66591 (9)	0.98982 (11)	0.0265 (4)	
C2	0.59828 (14)	0.72619 (10)	1.02190 (12)	0.0322 (4)	
H2	0.5820	0.7714	0.9908	0.039*	
C3	0.68734 (15)	0.72099 (12)	1.09875 (13)	0.0384 (4)	
H3	0.7314	0.7627	1.1202	0.046*	
C4	0.71225 (16)	0.65547 (12)	1.14417 (13)	0.0428 (5)	
H4A	0.7742	0.6516	1.1961	0.051*	
C5	0.64697 (17)	0.59559 (12)	1.11394 (14)	0.0436 (5)	
H5	0.6635	0.5507	1.1457	0.052*	
C6	0.55727 (15)	0.60058 (10)	1.03742 (13)	0.0349 (4)	
H6	0.5124	0.5592	1.0173	0.042*	
C7	0.43300 (14)	0.67069 (9)	0.90531 (11)	0.0281 (4)	
C8	0.44508 (14)	0.72594 (10)	0.84178 (11)	0.0305 (4)	
C9	0.38732 (17)	0.79023 (13)	0.81501 (14)	0.0450 (5)	
H9	0.3358	0.8015	0.8356	0.054*	
C10	0.4044 (2)	0.83823 (15)	0.75830 (17)	0.0616 (7)	
H10	0.3651	0.8824	0.7409	0.074*	
C11	0.47860 (19)	0.82192 (15)	0.72685 (15)	0.0557 (6)	
H11	0.4897	0.8546	0.6876	0.067*	
C12	0.53596 (16)	0.75824 (13)	0.75273 (13)	0.0441 (5)	
H12	0.5866	0.7469	0.7311	0.053*	
C13	0.52029 (15)	0.71046 (11)	0.81031 (12)	0.0355 (4)	
H13	0.5610	0.6669	0.8285	0.043*	
C14	0.34396 (13)	0.69218 (10)	0.93070 (11)	0.0277 (4)	
C15	0.10457 (13)	0.60501 (9)	0.90104 (11)	0.0263 (4)	
C16	−0.06058 (13)	0.58394 (9)	0.92138 (11)	0.0263 (4)	
C17	−0.08058 (14)	0.50973 (10)	0.90017 (11)	0.0284 (4)	
H17	−0.0326	0.4809	0.8872	0.034*	
C18	−0.17141 (14)	0.47832 (10)	0.89816 (11)	0.0305 (4)	
H18	−0.1858	0.4279	0.8831	0.037*	
C19	−0.24083 (13)	0.51957 (11)	0.91782 (12)	0.0317 (4)	
C20	−0.22088 (15)	0.59270 (11)	0.94035 (14)	0.0413 (5)	
H20	−0.2680	0.6209	0.9551	0.050*	
C21	−0.13118 (15)	0.62458 (11)	0.94123 (14)	0.0391 (5)	
H21	−0.1178	0.6752	0.9557	0.047*	
Cl2	0.85383 (5)	1.00209 (4)	0.11087 (5)	0.06182 (19)	
S2	0.40487 (4)	0.95519 (3)	0.18449 (3)	0.03181 (11)	
O4	0.09276 (10)	0.90499 (7)	0.15471 (8)	0.0336 (3)	
H4O	0.0816	0.9066	0.1998	0.050*	
O5	0.13097 (10)	0.75772 (7)	0.03205 (8)	0.0342 (3)	
N4	0.22880 (12)	0.85044 (9)	0.11506 (11)	0.0325 (3)	
H4N	0.2363 (17)	0.8894 (12)	0.1476 (14)	0.045 (6)*	
N5	0.31033 (12)	0.83735 (9)	0.09191 (11)	0.0362 (4)	
H5N	0.2991 (17)	0.8017 (13)	0.0518 (14)	0.046 (6)*	
N6	0.45659 (12)	0.86864 (9)	0.07879 (11)	0.0350 (4)	
H6N	0.4369 (17)	0.8333 (12)	0.0391 (14)	0.043 (6)*	
C22	−0.04212 (14)	0.84829 (9)	0.02956 (11)	0.0277 (4)	
C23	−0.04205 (16)	0.90432 (10)	−0.02701 (12)	0.0353 (4)	
H23	0.0192	0.9327	−0.0141	0.042*	
C24	−0.13058 (17)	0.91890 (11)	−0.10186 (13)	0.0418 (5)	
H24	−0.1298	0.9574	−0.1396	0.050*	
C25	−0.22013 (16)	0.87771 (11)	−0.12200 (13)	0.0406 (5)	
H25	−0.2807	0.8877	−0.1734	0.049*	
C26	−0.22073 (16)	0.82195 (11)	−0.06669 (13)	0.0391 (4)	
H26	−0.2819	0.7933	−0.0803	0.047*	
C27	−0.13221 (14)	0.80740 (10)	0.00893 (12)	0.0319 (4)	
H27	−0.1336	0.7691	0.0467	0.038*	
C28	0.05769 (14)	0.83534 (9)	0.11184 (11)	0.0272 (4)	
C29	0.04543 (14)	0.77964 (10)	0.17447 (11)	0.0306 (4)	
C30	0.10095 (18)	0.71399 (13)	0.19657 (15)	0.0487 (5)	
H30	0.1475	0.7019	0.1709	0.058*	
C31	0.0892 (2)	0.66601 (15)	0.25556 (18)	0.0653 (7)	
H31	0.1275	0.6213	0.2700	0.078*	
C32	0.0225 (2)	0.68284 (15)	0.29325 (15)	0.0581 (7)	
H32	0.0146	0.6497	0.3336	0.070*	
C33	−0.03331 (17)	0.74784 (13)	0.27265 (13)	0.0466 (5)	
H33	−0.0792	0.7596	0.2990	0.056*	
C34	−0.02215 (15)	0.79620 (11)	0.21312 (12)	0.0364 (4)	
H34	−0.0609	0.8408	0.1988	0.044*	
C35	0.14256 (14)	0.81022 (10)	0.08231 (11)	0.0285 (4)	
C36	0.39154 (14)	0.88456 (10)	0.11629 (11)	0.0293 (4)	
C37	0.55058 (14)	0.90486 (10)	0.08987 (11)	0.0290 (4)	
C38	0.62840 (16)	0.92071 (13)	0.17052 (13)	0.0420 (5)	
H38	0.6183	0.9107	0.2216	0.050*	
C39	0.72165 (16)	0.95127 (13)	0.17680 (14)	0.0473 (5)	
H39	0.7754	0.9623	0.2322	0.057*	
C40	0.73569 (15)	0.96547 (11)	0.10254 (13)	0.0366 (4)	
C41	0.65866 (14)	0.95032 (10)	0.02156 (12)	0.0322 (4)	
H41	0.6691	0.9603	−0.0294	0.039*	
C42	0.56583 (14)	0.92029 (10)	0.01565 (11)	0.0298 (4)	
H42	0.5119	0.9101	−0.0399	0.036*	

### Atomic displacement parameters (Å^2^)

**Table:**

	*U*^11^	*U*^22^	*U*^33^	*U*^12^	*U*^13^	*U*^23^
Cl1	0.0334 (3)	0.0594 (3)	0.0571 (3)	−0.0161 (2)	0.0271 (2)	−0.0117 (3)
S1	0.0354 (2)	0.0322 (2)	0.0347 (2)	−0.00651 (19)	0.0203 (2)	−0.00557 (18)
O1	0.0414 (8)	0.0347 (7)	0.0386 (7)	−0.0121 (6)	0.0261 (6)	−0.0140 (6)
O2	0.0322 (7)	0.0400 (7)	0.0472 (8)	−0.0083 (6)	0.0245 (6)	−0.0162 (6)
N1	0.0269 (8)	0.0357 (9)	0.0384 (8)	−0.0060 (7)	0.0196 (7)	−0.0086 (7)
N2	0.0273 (8)	0.0342 (9)	0.0427 (9)	−0.0073 (7)	0.0206 (7)	−0.0112 (7)
N3	0.0254 (8)	0.0269 (8)	0.0341 (8)	−0.0040 (6)	0.0149 (6)	−0.0040 (6)
C1	0.0246 (8)	0.0295 (9)	0.0313 (9)	−0.0011 (7)	0.0174 (7)	−0.0044 (7)
C2	0.0311 (10)	0.0328 (9)	0.0347 (9)	−0.0040 (8)	0.0158 (8)	−0.0008 (8)
C3	0.0307 (10)	0.0471 (12)	0.0375 (10)	−0.0097 (9)	0.0143 (9)	−0.0054 (9)
C4	0.0317 (10)	0.0582 (13)	0.0359 (10)	0.0033 (10)	0.0113 (9)	0.0056 (9)
C5	0.0469 (12)	0.0416 (11)	0.0457 (12)	0.0109 (10)	0.0228 (10)	0.0114 (9)
C6	0.0383 (11)	0.0307 (9)	0.0421 (10)	−0.0011 (8)	0.0232 (9)	−0.0011 (8)
C7	0.0285 (9)	0.0286 (9)	0.0319 (9)	−0.0054 (7)	0.0173 (8)	−0.0073 (7)
C8	0.0263 (9)	0.0387 (10)	0.0259 (8)	−0.0073 (8)	0.0102 (7)	−0.0034 (7)
C9	0.0371 (11)	0.0558 (13)	0.0453 (12)	0.0071 (10)	0.0204 (10)	0.0132 (10)
C10	0.0548 (15)	0.0659 (16)	0.0648 (16)	0.0168 (13)	0.0255 (13)	0.0318 (13)
C11	0.0503 (14)	0.0730 (17)	0.0456 (13)	−0.0039 (12)	0.0216 (11)	0.0225 (12)
C12	0.0372 (11)	0.0629 (14)	0.0365 (10)	−0.0121 (10)	0.0197 (9)	−0.0011 (10)
C13	0.0334 (10)	0.0429 (11)	0.0352 (10)	−0.0065 (9)	0.0193 (8)	−0.0045 (8)
C14	0.0246 (9)	0.0323 (9)	0.0285 (9)	−0.0037 (7)	0.0133 (7)	−0.0026 (7)
C15	0.0245 (8)	0.0294 (9)	0.0245 (8)	−0.0020 (7)	0.0098 (7)	0.0023 (7)
C16	0.0234 (8)	0.0302 (9)	0.0257 (8)	−0.0016 (7)	0.0107 (7)	0.0027 (7)
C17	0.0249 (8)	0.0298 (9)	0.0310 (9)	−0.0018 (7)	0.0121 (7)	−0.0002 (7)
C18	0.0281 (9)	0.0328 (9)	0.0297 (9)	−0.0061 (7)	0.0110 (7)	−0.0015 (7)
C19	0.0235 (9)	0.0410 (10)	0.0316 (9)	−0.0059 (8)	0.0124 (7)	0.0008 (8)
C20	0.0324 (10)	0.0395 (11)	0.0607 (13)	0.0004 (9)	0.0279 (10)	−0.0055 (10)
C21	0.0349 (10)	0.0312 (10)	0.0577 (13)	−0.0028 (8)	0.0256 (10)	−0.0059 (9)
Cl2	0.0482 (3)	0.0682 (4)	0.0875 (4)	−0.0305 (3)	0.0465 (3)	−0.0349 (3)
S2	0.0382 (3)	0.0311 (2)	0.0292 (2)	−0.0051 (2)	0.01702 (19)	−0.00241 (18)
O4	0.0416 (8)	0.0312 (7)	0.0379 (7)	−0.0097 (6)	0.0262 (6)	−0.0099 (5)
O5	0.0321 (7)	0.0342 (7)	0.0421 (7)	−0.0038 (6)	0.0213 (6)	−0.0107 (6)
N4	0.0285 (8)	0.0358 (9)	0.0408 (9)	−0.0061 (7)	0.0220 (7)	−0.0108 (7)
N5	0.0286 (8)	0.0410 (9)	0.0469 (10)	−0.0097 (7)	0.0236 (8)	−0.0147 (8)
N6	0.0321 (8)	0.0409 (9)	0.0383 (9)	−0.0131 (7)	0.0209 (7)	−0.0153 (7)
C22	0.0308 (9)	0.0255 (8)	0.0333 (9)	0.0023 (7)	0.0198 (8)	−0.0028 (7)
C23	0.0417 (11)	0.0321 (10)	0.0403 (10)	−0.0023 (8)	0.0254 (9)	−0.0006 (8)
C24	0.0561 (13)	0.0348 (10)	0.0388 (11)	0.0084 (10)	0.0240 (10)	0.0056 (8)
C25	0.0405 (11)	0.0437 (11)	0.0367 (10)	0.0119 (9)	0.0151 (9)	0.0004 (9)
C26	0.0325 (10)	0.0427 (11)	0.0410 (11)	0.0001 (9)	0.0142 (9)	−0.0042 (9)
C27	0.0342 (10)	0.0309 (9)	0.0346 (9)	−0.0002 (8)	0.0181 (8)	−0.0008 (8)
C28	0.0300 (9)	0.0256 (8)	0.0320 (9)	−0.0041 (7)	0.0187 (8)	−0.0048 (7)
C29	0.0290 (9)	0.0344 (10)	0.0292 (9)	−0.0082 (8)	0.0128 (8)	−0.0026 (7)
C30	0.0503 (13)	0.0486 (13)	0.0564 (13)	0.0089 (11)	0.0312 (11)	0.0164 (10)
C31	0.0720 (18)	0.0561 (15)	0.0774 (18)	0.0105 (13)	0.0405 (15)	0.0305 (13)
C32	0.0647 (16)	0.0634 (16)	0.0495 (13)	−0.0112 (13)	0.0269 (12)	0.0180 (12)
C33	0.0462 (12)	0.0634 (15)	0.0388 (11)	−0.0239 (11)	0.0263 (10)	−0.0105 (10)
C34	0.0359 (10)	0.0413 (11)	0.0362 (10)	−0.0119 (9)	0.0191 (9)	−0.0072 (8)
C35	0.0280 (9)	0.0307 (9)	0.0305 (9)	−0.0016 (7)	0.0158 (8)	0.0004 (7)
C36	0.0277 (9)	0.0332 (9)	0.0281 (9)	−0.0036 (7)	0.0127 (7)	0.0005 (7)
C37	0.0275 (9)	0.0297 (9)	0.0328 (9)	−0.0052 (7)	0.0156 (7)	−0.0037 (7)
C38	0.0361 (11)	0.0634 (14)	0.0296 (9)	−0.0134 (10)	0.0168 (8)	−0.0048 (9)
C39	0.0356 (11)	0.0711 (15)	0.0358 (10)	−0.0169 (11)	0.0155 (9)	−0.0188 (10)
C40	0.0312 (10)	0.0364 (10)	0.0499 (11)	−0.0102 (8)	0.0246 (9)	−0.0121 (9)
C41	0.0377 (10)	0.0293 (9)	0.0376 (10)	0.0009 (8)	0.0234 (8)	0.0019 (7)
C42	0.0298 (9)	0.0333 (9)	0.0261 (8)	0.0015 (8)	0.0114 (7)	−0.0005 (7)

### Geometric parameters (Å, °)

**Table:**

Cl1—C19	1.7407 (18)	Cl2—C40	1.7445 (19)
S1—C15	1.6791 (18)	S2—C36	1.6824 (19)
O1—C7	1.435 (2)	O4—C28	1.439 (2)
O1—H1O	0.8400	O4—H4O	0.8400
O2—C14	1.241 (2)	O5—C35	1.242 (2)
N1—C14	1.323 (2)	N4—C35	1.327 (2)
N1—N2	1.383 (2)	N4—N5	1.380 (2)
N1—H1N	0.89 (2)	N4—H4N	0.88 (2)
N2—C15	1.349 (2)	N5—C36	1.351 (2)
N2—H2N	0.81 (2)	N5—H5N	0.90 (2)
N3—C15	1.355 (2)	N6—C36	1.345 (2)
N3—C16	1.417 (2)	N6—C37	1.421 (2)
N3—H3N	0.88 (2)	N6—H6N	0.89 (2)
C1—C2	1.391 (2)	C22—C27	1.385 (2)
C1—C6	1.394 (3)	C22—C23	1.397 (3)
C1—C7	1.535 (2)	C22—C28	1.532 (3)
C2—C3	1.386 (3)	C23—C24	1.386 (3)
C2—H2	0.9500	C23—H23	0.9500
C3—C4	1.380 (3)	C24—C25	1.383 (3)
C3—H3	0.9500	C24—H24	0.9500
C4—C5	1.379 (3)	C25—C26	1.381 (3)
C4—H4A	0.9500	C25—H25	0.9500
C5—C6	1.387 (3)	C26—C27	1.392 (3)
C5—H5	0.9500	C26—H26	0.9500
C6—H6	0.9500	C27—H27	0.9500
C7—C8	1.530 (2)	C28—C29	1.525 (2)
C7—C14	1.537 (2)	C28—C35	1.545 (2)
C8—C9	1.387 (3)	C29—C30	1.390 (3)
C8—C13	1.398 (3)	C29—C34	1.394 (3)
C9—C10	1.389 (3)	C30—C31	1.385 (3)
C9—H9	0.9500	C30—H30	0.9500
C10—C11	1.389 (3)	C31—C32	1.372 (4)
C10—H10	0.9500	C31—H31	0.9500
C11—C12	1.374 (3)	C32—C33	1.380 (3)
C11—H11	0.9500	C32—H32	0.9500
C12—C13	1.389 (3)	C33—C34	1.393 (3)
C12—H12	0.9500	C33—H33	0.9500
C13—H13	0.9500	C34—H34	0.9500
C16—C21	1.388 (2)	C37—C38	1.380 (3)
C16—C17	1.393 (2)	C37—C42	1.387 (2)
C17—C18	1.390 (2)	C38—C39	1.390 (3)
C17—H17	0.9500	C38—H38	0.9500
C18—C19	1.379 (3)	C39—C40	1.375 (3)
C18—H18	0.9500	C39—H39	0.9500
C19—C20	1.378 (3)	C40—C41	1.377 (3)
C20—C21	1.386 (3)	C41—C42	1.383 (2)
C20—H20	0.9500	C41—H41	0.9500
C21—H21	0.9500	C42—H42	0.9500
			
C7—O1—H1O	109.5	C28—O4—H4O	109.5
C14—N1—N2	120.78 (16)	C35—N4—N5	120.94 (16)
C14—N1—H1N	123.6 (14)	C35—N4—H4N	123.9 (15)
N2—N1—H1N	115.2 (14)	N5—N4—H4N	114.8 (15)
C15—N2—N1	119.41 (16)	C36—N5—N4	120.32 (16)
C15—N2—H2N	123.1 (17)	C36—N5—H5N	123.2 (15)
N1—N2—H2N	117.5 (17)	N4—N5—H5N	115.4 (15)
C15—N3—C16	130.79 (15)	C36—N6—C37	128.29 (16)
C15—N3—H3N	115.2 (13)	C36—N6—H6N	116.9 (14)
C16—N3—H3N	114.0 (13)	C37—N6—H6N	114.7 (14)
C2—C1—C6	118.51 (17)	C27—C22—C23	118.62 (17)
C2—C1—C7	121.52 (16)	C27—C22—C28	123.20 (16)
C6—C1—C7	119.94 (16)	C23—C22—C28	118.18 (16)
C3—C2—C1	120.69 (18)	C24—C23—C22	120.55 (18)
C3—C2—H2	119.7	C24—C23—H23	119.7
C1—C2—H2	119.7	C22—C23—H23	119.7
C4—C3—C2	120.20 (19)	C25—C24—C23	120.42 (19)
C4—C3—H3	119.9	C25—C24—H24	119.8
C2—C3—H3	119.9	C23—C24—H24	119.8
C5—C4—C3	119.79 (19)	C26—C25—C24	119.42 (19)
C5—C4—H4A	120.1	C26—C25—H25	120.3
C3—C4—H4A	120.1	C24—C25—H25	120.3
C4—C5—C6	120.30 (19)	C25—C26—C27	120.40 (19)
C4—C5—H5	119.9	C25—C26—H26	119.8
C6—C5—H5	119.9	C27—C26—H26	119.8
C5—C6—C1	120.49 (18)	C22—C27—C26	120.58 (18)
C5—C6—H6	119.8	C22—C27—H27	119.7
C1—C6—H6	119.8	C26—C27—H27	119.7
O1—C7—C8	111.13 (14)	O4—C28—C29	110.30 (14)
O1—C7—C1	108.97 (14)	O4—C28—C22	108.47 (14)
C8—C7—C1	111.45 (14)	C29—C28—C22	113.96 (14)
O1—C7—C14	106.27 (13)	O4—C28—C35	105.77 (13)
C8—C7—C14	111.82 (15)	C29—C28—C35	110.80 (14)
C1—C7—C14	106.96 (13)	C22—C28—C35	107.16 (13)
C9—C8—C13	118.70 (18)	C30—C29—C34	118.53 (18)
C9—C8—C7	124.01 (17)	C30—C29—C28	122.81 (17)
C13—C8—C7	117.28 (17)	C34—C29—C28	118.63 (17)
C8—C9—C10	120.5 (2)	C31—C30—C29	120.7 (2)
C8—C9—H9	119.8	C31—C30—H30	119.6
C10—C9—H9	119.8	C29—C30—H30	119.6
C9—C10—C11	120.3 (2)	C32—C31—C30	120.3 (2)
C9—C10—H10	119.8	C32—C31—H31	119.9
C11—C10—H10	119.8	C30—C31—H31	119.9
C12—C11—C10	119.7 (2)	C31—C32—C33	120.1 (2)
C12—C11—H11	120.2	C31—C32—H32	119.9
C10—C11—H11	120.2	C33—C32—H32	119.9
C11—C12—C13	120.3 (2)	C32—C33—C34	119.9 (2)
C11—C12—H12	119.8	C32—C33—H33	120.1
C13—C12—H12	119.8	C34—C33—H33	120.1
C12—C13—C8	120.5 (2)	C33—C34—C29	120.5 (2)
C12—C13—H13	119.7	C33—C34—H34	119.8
C8—C13—H13	119.7	C29—C34—H34	119.8
O2—C14—N1	122.43 (16)	O5—C35—N4	123.06 (16)
O2—C14—C7	123.19 (15)	O5—C35—C28	123.25 (15)
N1—C14—C7	114.37 (15)	N4—C35—C28	113.68 (15)
N2—C15—N3	111.80 (15)	N6—C36—N5	112.18 (16)
N2—C15—S1	121.19 (13)	N6—C36—S2	125.65 (14)
N3—C15—S1	127.00 (13)	N5—C36—S2	122.17 (14)
C21—C16—C17	119.19 (16)	C38—C37—C42	119.58 (17)
C21—C16—N3	115.52 (15)	C38—C37—N6	122.87 (16)
C17—C16—N3	125.15 (16)	C42—C37—N6	117.42 (16)
C18—C17—C16	119.30 (17)	C37—C38—C39	119.87 (18)
C18—C17—H17	120.3	C37—C38—H38	120.1
C16—C17—H17	120.3	C39—C38—H38	120.1
C19—C18—C17	120.72 (17)	C40—C39—C38	119.69 (18)
C19—C18—H18	119.6	C40—C39—H39	120.2
C17—C18—H18	119.6	C38—C39—H39	120.2
C20—C19—C18	120.42 (17)	C39—C40—C41	121.18 (17)
C20—C19—Cl1	119.79 (15)	C39—C40—Cl2	119.50 (16)
C18—C19—Cl1	119.79 (14)	C41—C40—Cl2	119.31 (15)
C19—C20—C21	119.10 (18)	C40—C41—C42	118.90 (17)
C19—C20—H20	120.4	C40—C41—H41	120.5
C21—C20—H20	120.4	C42—C41—H41	120.5
C20—C21—C16	121.25 (18)	C41—C42—C37	120.77 (17)
C20—C21—H21	119.4	C41—C42—H42	119.6
C16—C21—H21	119.4	C37—C42—H42	119.6
			
C14—N1—N2—C15	−176.39 (17)	C35—N4—N5—C36	171.09 (17)
C6—C1—C2—C3	1.0 (3)	C27—C22—C23—C24	0.4 (3)
C7—C1—C2—C3	179.43 (16)	C28—C22—C23—C24	−179.29 (16)
C1—C2—C3—C4	0.5 (3)	C22—C23—C24—C25	−0.5 (3)
C2—C3—C4—C5	−1.5 (3)	C23—C24—C25—C26	0.2 (3)
C3—C4—C5—C6	1.0 (3)	C24—C25—C26—C27	0.3 (3)
C4—C5—C6—C1	0.5 (3)	C23—C22—C27—C26	0.0 (3)
C2—C1—C6—C5	−1.5 (3)	C28—C22—C27—C26	179.73 (16)
C7—C1—C6—C5	−179.97 (17)	C25—C26—C27—C22	−0.4 (3)
C2—C1—C7—O1	153.48 (15)	C27—C22—C28—O4	−130.14 (17)
C6—C1—C7—O1	−28.1 (2)	C23—C22—C28—O4	49.6 (2)
C2—C1—C7—C8	30.5 (2)	C27—C22—C28—C29	−6.9 (2)
C6—C1—C7—C8	−151.15 (16)	C23—C22—C28—C29	172.85 (15)
C2—C1—C7—C14	−92.03 (19)	C27—C22—C28—C35	116.09 (18)
C6—C1—C7—C14	86.35 (19)	C23—C22—C28—C35	−64.21 (19)
O1—C7—C8—C9	122.7 (2)	O4—C28—C29—C30	−119.9 (2)
C1—C7—C8—C9	−115.6 (2)	C22—C28—C29—C30	117.8 (2)
C14—C7—C8—C9	4.1 (2)	C35—C28—C29—C30	−3.1 (2)
O1—C7—C8—C13	−58.3 (2)	O4—C28—C29—C34	58.3 (2)
C1—C7—C8—C13	63.5 (2)	C22—C28—C29—C34	−64.0 (2)
C14—C7—C8—C13	−176.87 (15)	C35—C28—C29—C34	175.04 (15)
C13—C8—C9—C10	−0.2 (3)	C34—C29—C30—C31	0.1 (3)
C7—C8—C9—C10	178.8 (2)	C28—C29—C30—C31	178.3 (2)
C8—C9—C10—C11	0.8 (4)	C29—C30—C31—C32	−0.1 (4)
C9—C10—C11—C12	−0.6 (4)	C30—C31—C32—C33	−0.1 (4)
C10—C11—C12—C13	−0.3 (4)	C31—C32—C33—C34	0.3 (4)
C11—C12—C13—C8	0.9 (3)	C32—C33—C34—C29	−0.4 (3)
C9—C8—C13—C12	−0.6 (3)	C30—C29—C34—C33	0.1 (3)
C7—C8—C13—C12	−179.73 (17)	C28—C29—C34—C33	−178.10 (17)
N2—N1—C14—O2	−4.2 (3)	N5—N4—C35—O5	2.4 (3)
N2—N1—C14—C7	175.22 (16)	N5—N4—C35—C28	−176.98 (16)
O1—C7—C14—O2	171.68 (16)	O4—C28—C35—O5	−169.03 (16)
C8—C7—C14—O2	−66.9 (2)	C29—C28—C35—O5	71.4 (2)
C1—C7—C14—O2	55.4 (2)	C22—C28—C35—O5	−53.4 (2)
O1—C7—C14—N1	−7.7 (2)	O4—C28—C35—N4	10.4 (2)
C8—C7—C14—N1	113.68 (17)	C29—C28—C35—N4	−109.15 (17)
C1—C7—C14—N1	−124.05 (16)	C22—C28—C35—N4	125.97 (16)
N1—N2—C15—N3	179.07 (15)	C37—N6—C36—N5	−178.74 (18)
N1—N2—C15—S1	−1.2 (2)	C37—N6—C36—S2	2.3 (3)
C16—N3—C15—N2	−178.13 (16)	N4—N5—C36—N6	−171.84 (17)
C16—N3—C15—S1	2.2 (3)	N4—N5—C36—S2	7.1 (3)
C15—N3—C16—C21	−165.17 (18)	C36—N6—C37—C38	49.5 (3)
C15—N3—C16—C17	19.2 (3)	C36—N6—C37—C42	−134.7 (2)
C21—C16—C17—C18	0.9 (3)	C42—C37—C38—C39	−0.6 (3)
N3—C16—C17—C18	176.41 (16)	N6—C37—C38—C39	175.2 (2)
C16—C17—C18—C19	−0.7 (3)	C37—C38—C39—C40	0.0 (3)
C17—C18—C19—C20	−0.4 (3)	C38—C39—C40—C41	0.3 (3)
C17—C18—C19—Cl1	−179.70 (14)	C38—C39—C40—Cl2	−178.71 (18)
C18—C19—C20—C21	1.3 (3)	C39—C40—C41—C42	0.0 (3)
Cl1—C19—C20—C21	−179.38 (16)	Cl2—C40—C41—C42	179.06 (14)
C19—C20—C21—C16	−1.1 (3)	C40—C41—C42—C37	−0.7 (3)
C17—C16—C21—C20	0.0 (3)	C38—C37—C42—C41	1.0 (3)
N3—C16—C21—C20	−175.90 (19)	N6—C37—C42—C41	−175.05 (17)

### Hydrogen-bond geometry (Å, °)

**Table:**

*D*—H···*A*	*D*—H	H···*A*	*D*···*A*	*D*—H···*A*
O1—H1O···S2^i^	0.84	2.44	3.2242 (13)	156
N1—H1N···S1	0.89 (2)	2.44 (2)	2.9075 (16)	113.5 (17)
N2—H2N···O5^ii^	0.81 (2)	2.12 (2)	2.870 (2)	155 (2)
N3—H3N···O5^ii^	0.88 (2)	2.17 (2)	3.003 (2)	156.1 (18)
O4—H4O···S1^iii^	0.84	2.51	3.2707 (13)	151
N4—H4N···S2	0.88 (2)	2.50 (2)	2.9569 (16)	113.5 (17)
N5—H5N···O2^iv^	0.90 (2)	1.96 (2)	2.776 (2)	149 (2)
N6—H6N···O2^iv^	0.89 (2)	2.02 (2)	2.842 (2)	154 (2)

Symmetry codes: (i) *x*, −*y*+3/2, *z*+1/2; (ii) *x*, *y*, *z*+1; (iii) *x*, −*y*+3/2, *z*−1/2; (iv) *x*, *y*, *z*−1.

## References

[bb1] Bernstein, J., Davis, R. E., Shimoni, L. & Chang, N.-L. (1995). *Angew. Chem. Int. Ed. Engl.* **34**, 1555–1573.

[bb2] Bruker (1996). *XSCANS* Bruker AXS Inc., Madison, Wisconsin, USA.

[bb3] Bruker (2005). *SADABS* Bruker AXS Inc. Madison, Wisconsin, USA.

[bb4] Ergenç, N., Ilhan, E. & Ötük, G. (1992). *Pharmazie*, **47**, 59–60. 1608987

[bb5] Jalilian, A. R., Sattari, S., Bineshmarvasti, M., Shafiee, A. & Daneshtalab, M. (2000). *Arch. Pharm. Pharm. Med. Chem.* **333**, 347–354. 10.1002/1521-4184(200010)333:10<347::aid-ardp347>3.0.co;2-6 11092138

[bb6] John, A. D. (1998). *Lang’s Handbook of Chemistry, 4*, pp. 39-41. New York: McGraw-Hill.

[bb7] Kucukguzel, G., Kocatepa, A., DeClercq, E., Sahin, F. & Gulluce, M. (2006). *Eur. J. Med. Chem.* **41**, 353–359. 10.1016/j.ejmech.2005.11.005 16414150

[bb8] Sheldrick, G. M. (2008). *Acta Cryst.* A**64**, 112–122. 10.1107/S0108767307043930 18156677

[bb9] Shen, X., Shi, X., Kang, B., Liu, Y., Tong, Y., Jiang, H. & Chen, K. (1998). *Polyhedron*, **17**, 4049–4058.

[bb10] Singh, S., Husain, K., Athar, F. & Azam, A. (2005). *Eur. J. Pharm. Sci.* **25**, 255–262. 10.1016/j.ejps.2005.02.014 15911221

